# Supplementary material for: Uncovering the relationship between working memory and performance in the Jigsaw classroom
Source: PLoS One. 2025 Mar 18;20(3):e0319495. doi: 10.1371/journal.pone.0319495 (PMC11918379; doi:10.1371/journal.pone.0319495)

Supporting Information for

**Uncovering the relationship between working memory and performance in the Jigsaw classroom**

**This file includes:**

Sections S1 and S2, Figures S1-S3, Table S1

**S1. Detailed instructions for Jigsaw and control conditions**

**Procedure for Jigsaw group**

- The assignment to working group is randomized, by alphabetical order, to avoid the formation of strong relationship groups.

General Introduction: presentation of the new lesson about Critical Thinking to the students.

Instructions to students: *«*During this class, you will work into small groups and the functioning of your memory will be evaluated. You will develop your critical mind by learning about some reasoning biases, that we call “fallacious arguments”. You will discover them and learn how to debunk them. The final objective is to produce a collective work with your group, by creating new examples of fallacies. After this group activity, we will ask you again to take the Working Memory test, the same you took last time, and to individually answer some questions to check you properly understood the class. *»*

*1.*Play Video «Critical Thinking and Fallacious Arguments *» in whole class = 10 min.*

*2.*Formation of Jigsaw groups (4 students/group) and allocation of the material (1 handout/student).

Instructions to students: *«* Now, you must form the working groups. Each group must be composed of 4 students (must be adapted according to class size). Each student will receive its own handout with two fallacies to learn. For instance, in the Descartes handout, there are fallacies named *« slippery-slope argument »*and*« appeal to ignorance».*You will get 5 minutes to read the contents of your handout. In this part, there is, therefore, absolutely no interaction with your classmates. » = 5 min.

3.      Formation of the Expert groups (5 students/group).

The professor identifies the students who share the same handout and gather them into appropriate expert groups. Clone expert groups which share the same handout, when required). Instructions to students: « Into your own expert group, you must share each other information about your handout, to become the expert of your subject. You can take notes and create new fallacies together. The goal is to understand very well each of the two fallacies of your handout, to be able to explain to your initial groupmates, once you’ll be back in your homegroup. = 15 min.

1. Back to Homegroup

Instructions to students: « The expert phase is now over. Each student returns his/her initial group. The objective is to explain the fallacies of your handout to your group members. You can use the notes you took during the expert phase. Be careful, speaking time of each student cannot exceed 8 minutes. Each student must imperatively get his/her turn to speak because every member in the group must have known about all the 8 fallacies form the 4 handouts. = 30 min (± 8 min/pers).

1. Distribution of the tablets for the Working Memory test.

Students use the same ID they created during the baseline. The test is taken individually. = 10 min

1. Collective production task.

Every group is randomly assigned a topic among a 14 topics list. Each group is randomly assigned an envelope with 4 sorts of fallacies to produce.

Instructions to students: «The objective of this work is to collectively produce, as a group, new examples of fallacious arguments, according to the topic you choose and the sort of fallacies your group have been assigned to. Play the game, and propose new fallacies which are different than the ones you have been expert of. It’s in your interest, and if your experts made the job well, you can create new ones. Those new examples must be returned on a separate sheet, that every group must submit ». = 20 min.

7. Students get their tablets back to fulfil the NASA-RTLX questionnaire and the individual Quiz. = 15 min.

**Procedure for Control group**

- Pedagogical contents must not be divided among a working group.
- The assignment to working group is randomized, by alphabetical order, to avoid the formation of strong relationships groups.

General Introduction: presentation of the new lesson about Critical Thinking to the students.

Instructions to students: *«*During this class, you will work into small groups and the functioning of your memory will be evaluated. You will develop your critical mind by learning about some reasoning biases, that we call “fallacious arguments”. You will discover them and learn how to debunk them. The final objective is to produce a collective work with your group, by creating new examples of fallacies. After this group activity, we will ask you again to take the Working Memory test, the same you took last time, and to individually answer some questions to check you properly understood the class. *»*

1. Play Video «Critical Thinking and Fallacious Arguments *» in whole class = 10 min.*
2. Formation of the working group

Ask for 4 students per group (5 or 6 max). Distribution of the material: a handout composed of the 8 fallacies to learn.

Instructions to students: « Now, you must form the working groups. Each group must be composed of 4 students (must be adapted according to class size). I will distribute the handout and your objective is to work on the contents, with your group. You have 40 minutes to task about and understand the entirety of the fallacies, because after you’ll be asked to create new examples with your own group ». Reading = 20 minutes, Group discussion = 40 min.

1. Distribution of the tablets for the Working Memory test.

Students use the same ID they created during the baseline. The test is taken individually. = 10 min.

1. Collective production task.

Every group is randomly assigned a topic among a 14 topics list. Each group is randomly assigned an envelope with 4 sorts of fallacies to produce.

Instructions to students: «The objective of this work is to collectively produce, as a group, new examples of fallacious arguments, according to the topic you choose and the sort of fallacies your group have been assigned to. Those new examples must be returned on a separate sheet, that every group must submit ». = 20 min.

1. Students get their tablets back to fulfil the NASA-RTLX questionnaire and the individual Quiz. = 15 min.

**S2. Scoring details for the collective production task**

Although the jigsaw procedure does not call for group evaluation but individual one (e.g., quiz), we decided to assess our collective production task to check for equality of the cooperative settings, and students’ success to this creative task. The collective production task, inspired by Ennis-Weir critical thinking essay test (Ennis & Weir, 1985), was employed as an incentive to work in small groups in both experimental and control conditions. Groups (N=80) were asked for the creation of 4 examples of logical fallacies. The goal of this group activity was to defend a quirky statement (e.g., ‘retirees are responsible for global warming’), by creating new logical fallacies from randomly assigned categories (e.g., slippery slope). To succeed the task, novel arguments had to follow the rules and the logics of the assigned category. Each success was coded 1, each failure or missing argument was coded 0 (see table S3, for descriptive statistics), and the final score could range from 0 to 4. Responses were scored by two co-authors (blind to conditions), who cooperated and had discussion and agreement on each item. As can be seen in Table S3, descriptive statistics indicate similar collective performances in both learning conditions. No further analysis was conducted to test for the moderating role of individual WMC scores on this collective task due to risk of committing ecological fallacy. Even aggregating individual WMC scores within each of the 80 groups of students would probably not capture the “group working memory”. In the absence of any available group working memory task, future research on Jigsaw classroom could compare individual and group performances by using the nominal group method.

**Figure S1**

***
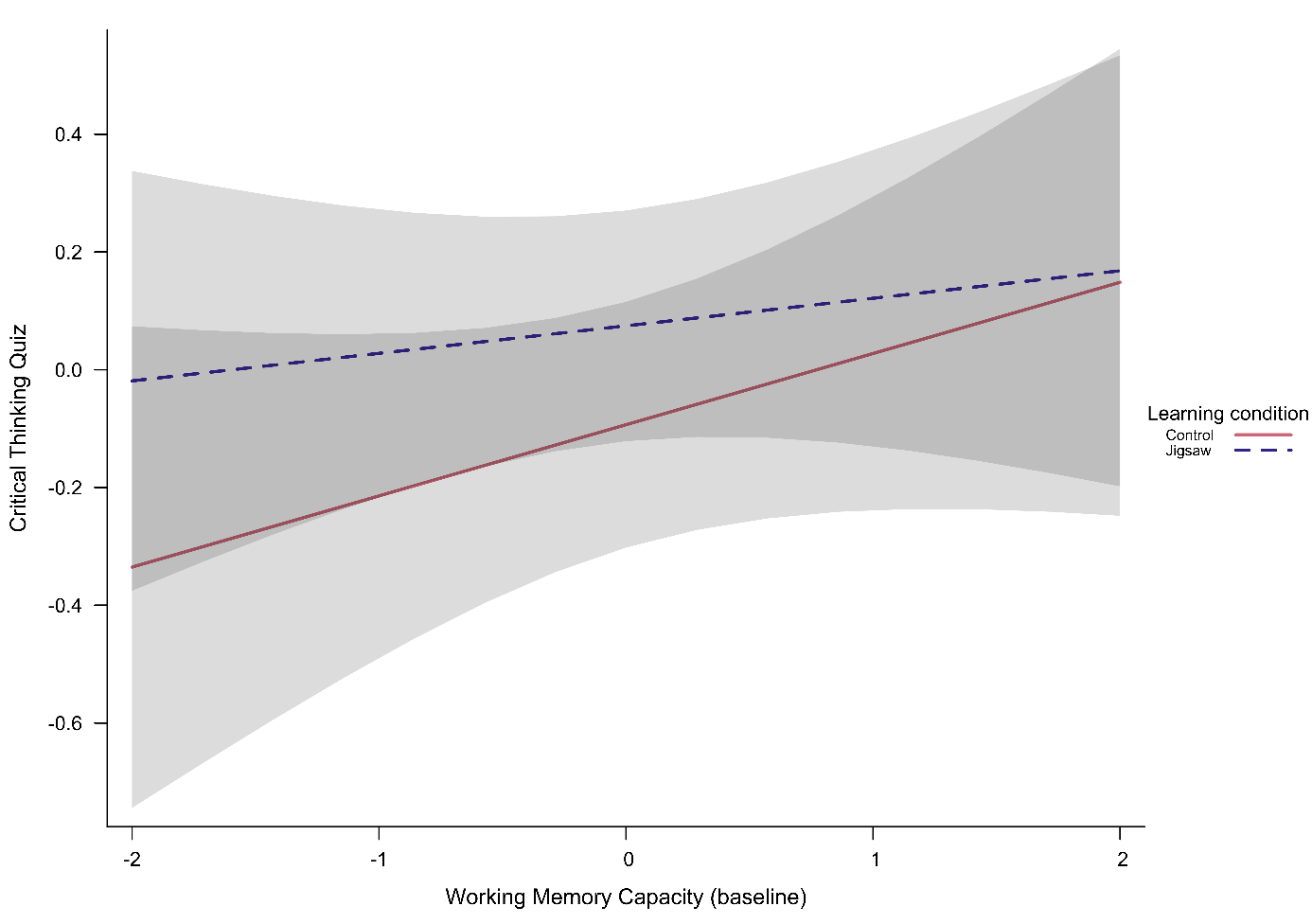
****Moderation model for Learning Condition and Working Memory Capacity (baseline) on individual quiz performance*

*Note.* Error bands indicate 95% confidence intervals. Y-axis is truncated for greater visibility.

**Figure S2**

*Interaction effect between Learning Condition and Working Memory Capacity (absolute scoring) on raw scores of individual quiz performance*

**
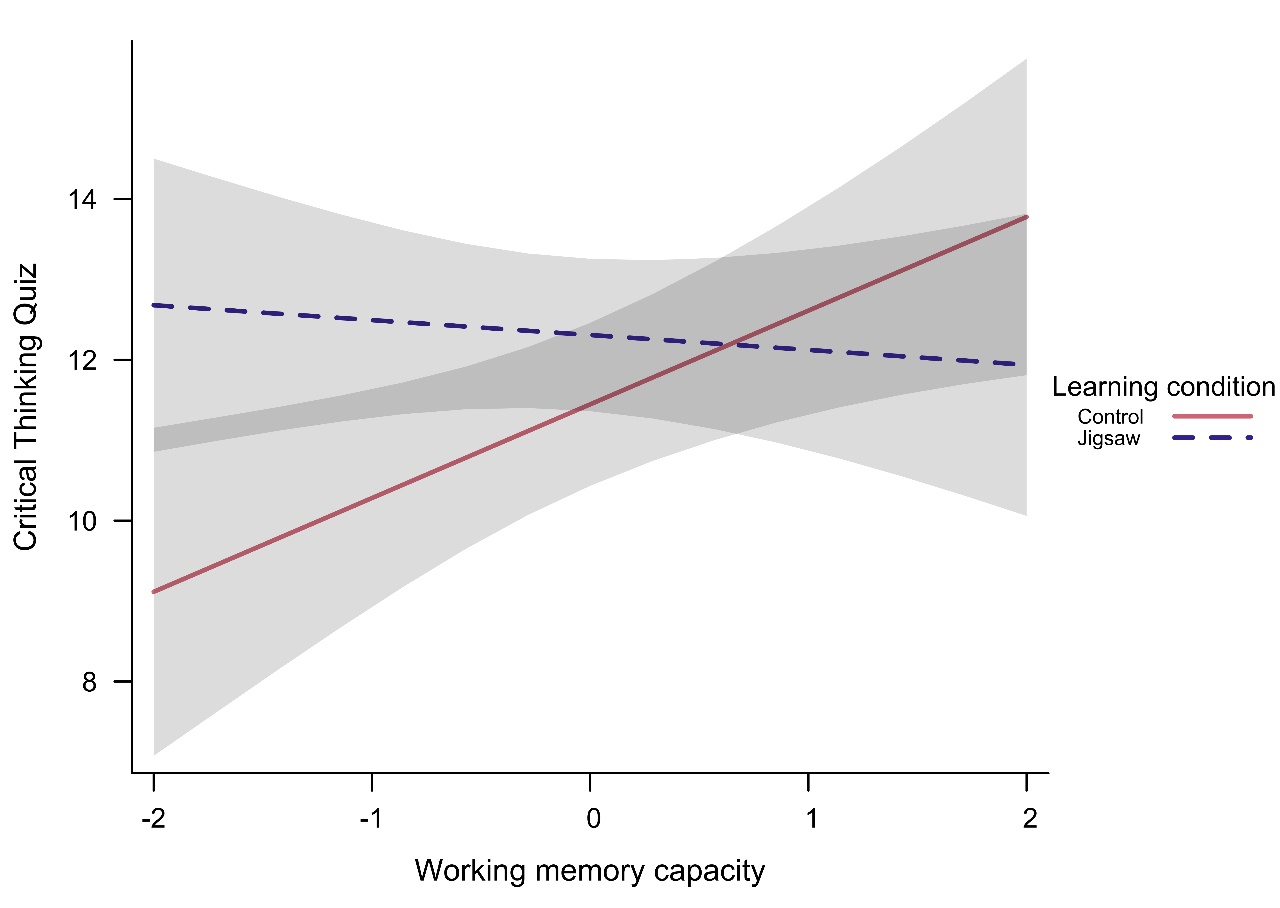
**

*Note.* Error bands indicate 95% confidence intervals. Y-axis is truncated for greater visibility.

|  | **Quiz** | |  | |
| --- | --- | --- | --- | --- |
| *Predictors* | *Estimates* | *CI* | *SE* | *p* |
| (Intercept) | -1.17 | [-2.06 – -0.28] | .045 | 0.010 |
| Symmetry Rate | **.012** | **[0.00 – 0.02]** | **.005** | **0.014** |
| Condition [Jigsaw] | .190 | [-1.29 – 1.66] | .747 | 0.805 |
| Symmetry Rate X Condition | -0.000 | [-0.02 – 0.02] | .008 | 0.938 |
| **Random Effects** | | | |  |
| σ^2^ | 0.82 | | |  |
| τ_00_ _group_ | 0.16 | | |  |
| ICC | 0.16 | | |  |
| N _group_ | 80 | | |  |
| Observations | 342 | | |  |
| Marginal R^2^ / Conditional R^2^ | 0.031 / 0.186 | | |  |

**Table S1. Moderation analysis with the symmetry processing task**

Moderation analysis (lmm) performed on the individual critical thinking performance (quiz). The tested interaction between the symmetry rate component (processing task of the complex span task) and condition is reported.

**Figure S3. Moderation analysis with the symmetry processing task**

Linear Mixed Model for the Symmetry Rate (processing task), the Condition and their interaction on the individual critical thinking performance (quiz).


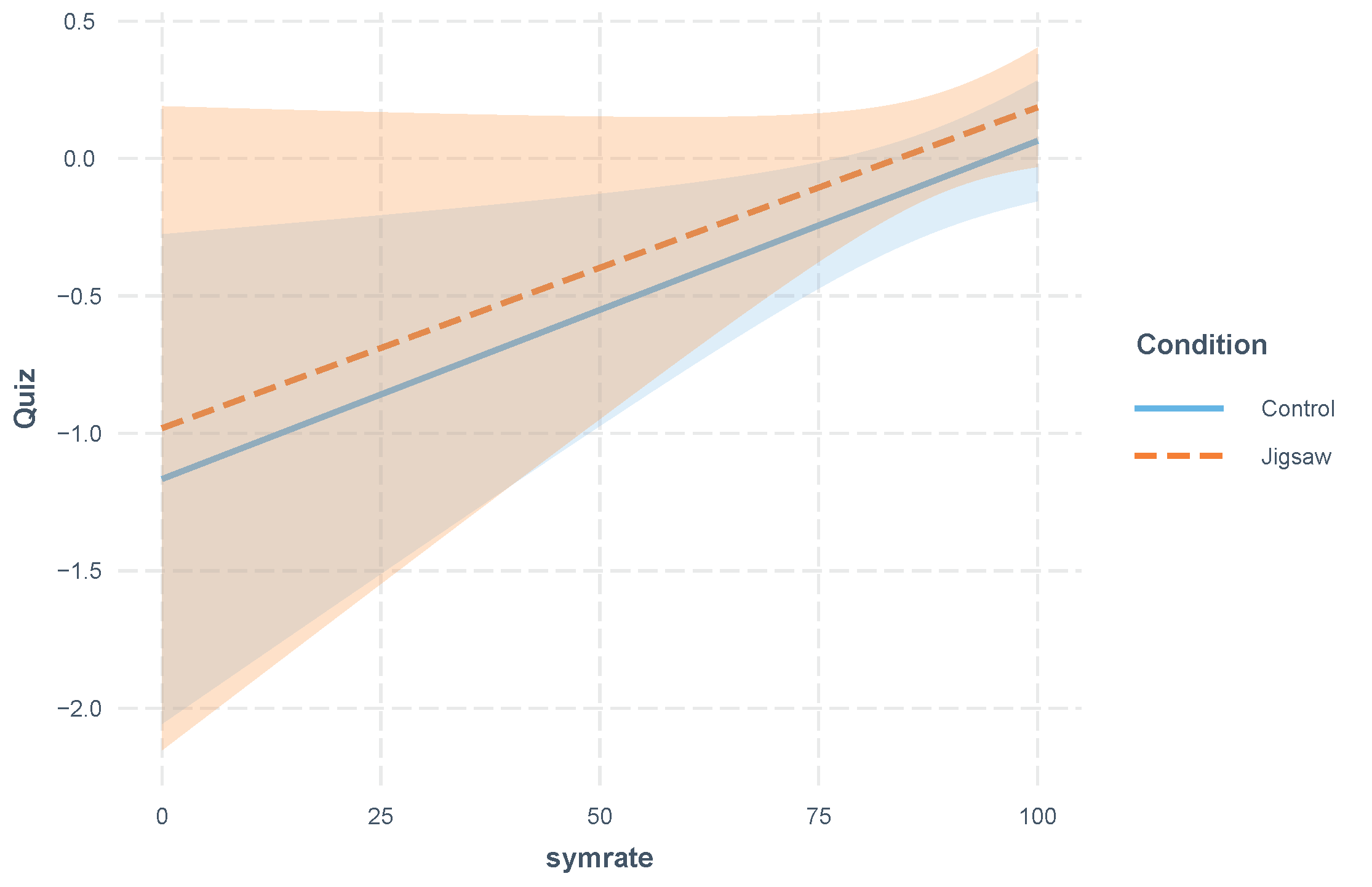

Supplement: S1 — (DOCX) [file pone.0319495.s001.docx]
