# Supplementary material for: Uncovering the relationship between working memory and performance in the Jigsaw classroom
Source: PLoS One. 2025 Mar 18;20(3):e0319495. doi: 10.1371/journal.pone.0319495 (PMC11918379; doi:10.1371/journal.pone.0319495)
Supplement: Attachment — Submitted filename: Response_to_Reviewers.docx [file pone.0319495.s003.docx]

**1. Is the manuscript technically sound, and do the data support the conclusions?**

The manuscript must describe a technically sound piece of scientific research with data that supports the conclusions. Experiments must have been conducted rigorously, with appropriate controls, replication, and sample sizes. The conclusions must be drawn appropriately based on the data presented.

Reviewer #1: No

Reviewer #2: Yes

**2. Has the statistical analysis been performed appropriately and rigorously?**

Reviewer #1: No

Reviewer #2: Yes

**3. Have the authors made all data underlying the findings in their manuscript fully available?**

The PLOS Data policy requires authors to make all data underlying the findings described in their manuscript fully available without restriction, with rare exception (please refer to the Data Availability Statement in the manuscript PDF file). The data should be provided as part of the manuscript or its supporting information, or deposited to a public repository. For example, in addition to summary statistics, the data points behind means, medians and variance measures should be available. If there are restrictions on publicly sharing data—e.g. participant privacy or use of data from a third party—those must be specified.

Reviewer #1: Yes

Reviewer #2: Yes

**4. Is the manuscript presented in an intelligible fashion and written in standard English?**

PLOS ONE does not copyedit accepted manuscripts, so the language in submitted articles must be clear, correct, and unambiguous. Any typographical or grammatical errors should be corrected at revision, so please note any specific errors here.

Reviewer #1: Yes

Reviewer #2: Yes

**5. Review Comments to the Author**

Please use the space provided to explain your answers to the questions above. You may also include additional comments for the author, including concerns about dual publication, research ethics, or publication ethics. (Please upload your review as an attachment if it exceeds 20,000 characters)

**Author’s response**: We would like to thank both the Reviewers for their comments as well as their suggestions for improving our manuscript. We would also like to thank the Editor for their interest in our manuscript. You will find a point-by-point response to the issues raised by the Reviewers. Responses are written in blue font in our letter and in the revised version of the manuscript.

**Reviewer #1**: Thank you for the opportunity to review the manuscript entitled: "Uncovering the relationship between working memory and performance in the cooperative Jigsaw classroom”.

Currently, in the education more than teaching processes and the teacher’s work in the classroom, it is the learning processes through which students achieve their proposed objectives in each subject that capture most interest. In accordance with this new approach centered on learning, the use of methodologies based on active constructive learning such as cooperative learning. This methodology enables students to acquire basic skills and increases their motivation to participate actively in the learning process. These days, learning methods based on cooperation are more and more widely used with the aim of encouraging teamwork, allowing students to learn to work as part of a team, improving performance and learning and developing interpersonal skills.

The paper examined whether working memory capacity mediated or moderated the effects of Jigsaw classroom on individual performance. Also, the findings offer insight into the potential cognitive mechanisms implied in the success of the Jigsaw method and provide new recommendations for educators on how to redeem the deficit of low working-memory capacity students on performance.

**R1:** Introduction: Cooperative learning has been widely investigated since the 1970s, when the first studies on specific implementations emerged. According to Gillies (2016), several metha-analyses have provided ample evidence of the effectiveness of cooperative learning on students’ outcomes: learning and performance. Some investigations have focused mainly on issues related to the efficacy of cooperative learning and the mediating mechanisms involved. The goal of these investigations revolves around two axes. The first one is the nature and quality of the interactive process. The second axis refers to prior factors that condition the efficacy of cooperative learning.

The authors suggest that taking into account potential moderating and mediating cognitive variables would help to understand Jigsaw mixed findings by specifying for whom and how the Jigsaw method can benefit (or not) to academic achievement. Given the Jigsaw method's reliance on resource interdependence, investigating working memory (WM) becomes pertinent.

Why can cognitive variables such as working memory be mediators or moderators? There are many variables such as learning goals, self-concept, anxiety, expectations, attitudes towards teamwork, social skills, cognitive style…, that can be mediators or moderators. The introduction does not provide sufficient background. It lacks a robust conceptual framework.

**Author's response**: We agree with the reviewer that the above variables may be relevant mediators and moderators of cooperative learning on achievement. Indeed, a recent review (Authors et al., 2024) found that learning goals, self-concept, and attitudes towards Jigsaw classroom have received considerable interest from practitioners and academics. However, little or no work to date, has explored the relationships between individuals' cognitive abilities and cooperative learning in the classroom. Given that working memory capacity is a well-established predictor of academic performance and learning capacity during a course, it was critical to explore this "third variable" in the context of the Jigsaw classroom. The Jigsaw method, which uniquely relies on resource interdependence and structured peer teaching, demands considerable cognitive engagement from students. As detailed in the section *Inside the Black Box of Jigsaw Classroom*, we argue that examining WM may thus enable us to address a notable gap in cooperative learning research by illuminating how cognitive resources interact with this specific instructional approach. Our focus on working memory aligns with current research on cognitive load in collaborative learning (Zambrano et al., 2019; Kirschner et al., 2018), contributing to a deeper understanding of how cooperative methods may differentially benefit learners based on cognitive capacity.

We have, hopefully, made our explanation clearer in the manuscript on page 6.

**R1:** Participants: The participants were not chosen at random. The number of participants from a university is not representative and directly affects external validity.

**Author’s response**: We acknowledge the reviewer’s point regarding the non-random nature of our sample and the limitations it may pose for generalizing findings beyond the university context. As with many studies conducted among university students, our sample may not be representative of the general population.

However, our research was designed as a field study to examine the effects of the Jigsaw method among undergraduate students within a natural classroom setting, rather than in a controlled laboratory environment. A random sample of participants from the general population would not have met our research objectives, which were to test the effectiveness of this cooperative learning approach in a university context where it is commonly applied.

Moreover, we tested a very large sample of students (n = 342 *participants*). This sample size falls within the higher range of samples commonly used in this area of research. On average, studies conducted with Jigsaw classroom have used relatively smaller sample sizes. Data from a recent systematic review (Authors et al., 2024) showed that of the 69 studies included in the review, the average sample size was 119.37 *participants*. This means that our sample size is 187% larger than the average reported in the review and is positioned in the 97^th^ percentile of sample sizes. Our sample is thus notably larger, enhancing the robustness and reliability of our findings within the university context.

Finally, we made sure that different disciplines were represented by having classes in biology, statistics and economics. A total of 12 classes were enrolled in the study, from different sites of the faculty. This diversity contributes to the heterogeneity of our sample and supports the ecological validity of our findings within higher education settings.

For clarity and transparency purposes, we have inserted an explanation about our sample on page 8 of the manuscript: “We used a large convenience sample of first-year undergraduate science students (N = 342; 12 classes ranging from 20 to 33 students). This sample was particularly appropriate for testing the effects of the Jigsaw classroom in higher education settings, where the method is commonly used. Students voluntarily participated in the study as part of a new general university course, “Methodology”, which focused on learning strategies”.

**R1:** Instruments: The instruments must always display two important qualities: reliability and validity. The authors should have calculated the reliability coefficients for their sample (NASA-RTLX scale) and should have calculated McDonald's Omega, Composite Reliability (CR) and Average Variance Extracted (AVE) of the subscales (effort, frustration, mental demand, physical demand, temporal demand, and task success). Cronbach's Alpha is conditioned by the number of items and the number of alternative responses, so it is necessary to use other alternative reliability indices, such as McDonald's Omega which is calculated through factorial loads and are measured more accurate reliability.

**Author’s response**: We thank the Reviewer for their comments and their request to provide the psychometric properties for the NASA-RTLX scale. We agree with the Reviewer when they say Cronbach’s alpha would not be an appropriate reliability parameter. Indeed, assessing internal consistency of this questionnaire does not align with the scale design, as the NASA-RTLX measures six different types of workloads, not a unidimensional construct. Calculating the McDonald’s Omega, for similar reasons, would not provide useful information about reliability, as the six dimensions of the NASA-RTLX are not conceptually expected to correlate as highly as items on a unidimensional scale.

A more appropriate measure of reliability for the NASA-RTLX would be test-retest reliability or an intra-class correlation coefficient (ICC) to assess the stability of responses across time for each dimension independently, as reported in the literature (e.g. Devos et al., 2020; Rubio et al., 2004). However, such an analysis was beyond the scope of the present study. In line with existing studies using the NASA-RTLX, we followed established practice by focusing on each dimension individually without conducting an internal consistency analysis, as this would misrepresent the scale's design.

This information is now presented on page 10: “Following established practice and the recommendations of Byers et al. (1989), we analyzed each subscale individually and did not conduct an internal consistency analysis, as this would misrepresent the scale’s design”. The reliability parameters of the working memory measures were also reported in Table 1, on page 32. This is now indicated clearly on page 14: “Table 1 presents dependent variable correlations and reliability coefficients (Cronbach’s α), and Table 2 reports samples descriptive statistics and mean difference effect sizes (Cohen’s d)”.

**R1:** Did the “task success-perfomance” scale use range from 1 from 1 (very low) to 21 (very high)?

**Author’s response** : Yes, as with the five other NASA-TLX subscales, the performance subscale used a 21-point scale (from 1-very low to 21-very high). This item specifically asked participants, “Do you believe you have successfully completed the tasks required for this assignment?” (in French in the study). This approach aligns with the standard use of NASA-TLX scales, ensuring consistency across workload dimensions.

For clarity we have changed the name of this subscale to “performance” (see on page 10) which is congruent with the original publication (Byers et al., 1989).

**R1:** Results

**R1:** Effect sizes differences should have been calculated using Cohen´s d or Hedge´s g. (Table 2)

**Author’s response:** We thank the Reviewer for this valuable suggestion and have now reported Cohen’s d to quantify the differences between the Jigsaw and control groups. This change is now indicated on page 15 and appears in Table 2, on page 33.

Additionally, we have included an explanation of our approach to reporting effect sizes in mixed models on page 14 “Finally, considering the challenges discussed in the literature regarding standardized effect sizes in linear mixed models—particularly the complications introduced by variance partitioning (Pek & Flora, 2018)—we report effect sizes using standardized beta coefficients. This approach, common in mixed model analyses, ensures statistical rigor and reproducibility while appropriately addressing the nested data structure and random effects (Lorah, 2018). Differences between the Jigsaw and control groups are also provided in Table 2 in the form of Cohen’s d.

References

Lorah, J. (2018). Effect size measures for multilevel models: Definition, interpretation, and TIMSS example. *Large-scale assessments in education*, *6*(1), 1-11.

Pek, J., & Flora, D. B. (2018). Reporting effect sizes in original psychological research: A discussion and tutorial. *Psychological methods*, *23*(2), 208.

**R1:** Mediation Model Analysis (Table 3). The mediation model must meet the requirements to conduct a simple mediation analysis: significant relations between the independent (LC) and the dependent (Quiz) variables (c= total effect), between the independent (LC) variable and the mediator (VMC, a), and between the mediator (VMC) and the dependent variable (Quiz, b). Additionally, the b score is larger than c´ (direct effect), and c` is smaller than c.

In this study there are no significant relations (a: LC-VMC, β=- .044, p>0.05; c: LC-Quiz, β=- .082, p>0.05). If the conditions were not met, why did the authors perform the mediation analysis? It makes no sense.

**Author’s response**: We appreciate the reviewer's feedback and understand the concern about our approach to mediation analysis. We recognise that the Reviewer's comments are based on the classical mediation framework proposed by Baron and Kenny (1986), which requires certain conditions to be met before conducting a mediation analysis. However, our analysis is based on a more contemporary theoretical approach, namely the *index approach* to mediation, proposed by Hayes (2013, 2017, 2022). Unlike *the causal steps* approach of Baron and Kenny, Hayes’ approach focuses on testing the indirect effect directly, without requiring that all individual paths be significant. This perspective, which has been widely adopted, is considered both statistically robust and flexible. It allows for mediation to be established through an indirect pathway that can exist independently of a significant total effect (Hayes & Rockwood, 2020). This is particularly relevant in cases where complex mechanisms may still operate through an indirect pathway, which aligns with our theoretical framework for exploring underlying cognitive mechanisms in the Jigsaw method. This modern approach recognises that meaningful mediation can occur in the absence of significant direct relationships, thereby extending the applicability of mediation analysis to a wider range of data structures. We hope this clarifies our methodological choice and its alignment with current statistical practice.

Furthermore, in support of transparency and reproducibility in science, we chose to fully report our argumentation process - from the development of our theoretical hypothesis of mediation to the detailed presentation of our statistical results. We believe that sharing our mediation analysis, even in cases where results were not statistically significant, is essential in the context of the current reproducibility crisis (Franco et al., 2014). Our aim was to publish our full methodological approach, including cases where the data did not support our hypotheses, as we believe that such findings may still be valuable for future research in this field. This approach is also consistent with PLOS ONE's ethical guidelines, which support the publication of negative, null and inconclusive results. We hope that the Reviewer appreciates this concern for transparency and the value we see in contributing our findings to the wider scientific conversation, regardless of statistical significance.

For clarity this was inserted in the main text on page 13: “Our mediation analysis was based on the index approach to mediation proposed by Hayes (2022), that focuses on testing the indirect path directly, without requiring that all individual paths be significant.”

References

Franco, A., Malhotra, N., & Simonovits, G. (2014). Publication bias in the social sciences: Unlocking

the file drawer. *Science*, *345*(6203), 1502-1505.

Hayes, A. F. (2022)*. Introduction to mediation, moderation, and conditional process analysis: A*

*regression-based approach* (3rd Ed.) New York: The Guilford Press. (1st and 2nd editions published in 2013 and 2018).

Hayes, A. F., & Rockwood, N. J. (2020). Conditional process analysis: Concepts, computation, and

advances in the modeling of the contingencies of mechanisms. *American Behavioral Scientist*, 64(1), 19-54.

**R1:** Moderation Model Analysis (Table 4). In this study there are no significant relationships between the independent (LC) and dependent (Quiz) variable (β=- .174, [-.102, .456]). It makes no sense to look for a moderator variable between two variables that are not significantly related.

We do not know conditional effects of the focal predictor at values of the moderator.

**Author’s response**: We appreciate the Reviewer’s insight regarding our moderation analysis. Our decision to test for moderation aligns with a contemporary statistical perspective (Hayes, 2022), which holds that a significant overall relationship between the independent variable (Learning Condition) and the dependent variable (Quiz) is not a prerequisite for examining moderation. When a moderator significantly influences the IV-DV relationship, analyzing conditional effects can reveal valuable insights that may not appear in the aggregate effect alone.

Additionally, Simpson’s Paradox highlights the importance of examining subgroup interactions, even when the overall relationship between the independent and dependent variables is weak or non-significant. Simpson’s Paradox illustrates how patterns within subgroups can be masked or even reversed in aggregate data (Wagner, 1982).

In our study, this approach indeed revealed a significant relationship between the Jigsaw group and Quiz performance at specific values of the moderator (the WMC). This valuable relationship would have remained undetected without a moderation analysis, supporting our hypothesis regarding the influence of cognitive abilities on learning outcomes in cooperative settings.

This explanation is now reported on pages 13-14: “For moderation, we followed Hayes' (2022) approach, which argues that a significant overall relationship between the independent and dependent variables is not necessary to test for moderation.”

Reference

Wagner, C. H. (1982). Simpson's paradox in real life. *The American Statistician*, *36*(1), 46-48.

**R1:** References: Lack of up-to-date references. The authors only present three current references (last five years 2020-2024). Thank you.

**Author’s response :** We thank the Reviewer for this comment. We have now included more recent articles on the effects of the Jigsaw classroom. Additionally, a reference cited in the main text (Friso-van den Bos & van de Weijer-Bergsma) was previously missing from the references section and has now been added. The year for Stanczak et al.’s paper has been corrected (it is 2022, not 2021). All references are now properly cited both in the main text and in the references section. Please see the changes we made on page 4 and in the references section.

“However, and despite its popularity, the Jigsaw method yields a mix of positive, null, and negative effects on both academic achievement (e.g., Law, 2011; Roseth et al., 2019; Moskowitz et al., 1983) and psychosocial variables such as self-regulation and self-perceptions (e.g., Riant et al., 2024; Rudmann et al., 2024) (for a comprehensive review see Authors, 2024).”

References

Friso-van den Bos, I., & van de Weijer-Bergsma, E. (2020). Classroom versus individual working memory assessment: Predicting academic achievement and the role of attention and response inhibition. *Memory*, 28(1), 70-82. https://doi.org/10.1080/09658211.2019.1682170

Hayes, A. F. (2022). *Introduction to mediation, moderation, and conditional process analysis: A regression-based approach* (3rd Ed.) New York: The Guilford Press. (1st and 2nd editions published in 2013 and 2018).

Riant, M., de Place, A. L., Bressoux, P., Batruch, A., Bouet, M., Bressan, M., ... & Pansu, P. (2024). Does the Jigsaw method improve motivation and self-regulation in vocational high schools?. *Contemporary Educational Psychology*, *77*, 102278. https://doi.org/10.1016/j.cedpsych.2024.102278

Rudmann, O., Batruch, A., Visintin, E. P., Sommet, N., Bressoux, P., Darnon, C., ... & Butera, F. (2024). Cooperative learning reduces the gender gap in perceived social competences: A large-scale nationwide longitudinal experiment. *Journal of Educational Psychology*, *116*(6), 903. <https://doi.org/10.1037/edu0000870>

**Reviewer #2:** It is an interesting and thought provoking research. I wonder of whether the relationship between condition (Jigsaw or control) and critical thinking performance is moderated specifically by WMC. Both developmental cognitive psychology (e.g., Fry & Hale, 2000) and cognitive aging literature (e.g., Verhaeghen, 2011 -metaanalysis of over 100 studies; Salthouse, 1996) indicate that the processing speed is related directly and by mediation of working memory to complex cognition. The ASSPAN measure of working memory consist of the two tasks: The storage task and the processing task. As I understand the storage task was used as the principal measure of WMC in the moderating analysis (Table 4). I wonder of whether the same results would be obtained when Symmetry Rate Test instead of Absolute Span Test (this symmetry judgment task seems to be closely related to the processing speed task) was applied as moderating variable? Interestingly (see Table 1) the correlations between Critical Thinking Quiz and both Absolute Span Test and Symmetry Rate Test were significant and almost identical.

**R2:** Therefore I'd suggest that Authors discuss the possibility that the Jigsaw Classroom could enhance performance not only students with low working memory capabilities but also students with slow processing speed.

**Author’s response:** We thank the Reviewer for their suggestions and the references provided. Indeed, in complex working memory span tasks, the processing task is a central element, predictive of the recall and accounting for the variance of higher-order cognition (Unsworth, 2009).

It is important to note that this component was voluntarily not tested as a potential moderator of the learning condition on the critical thinking quiz, because the symmetry span was serving for our screening criteria. As we explain on page 9 of the manuscript, participants failing below 80% on the processing component, either on the WM baseline and/or the WM test, were excluded from data analysis (see Conway, 2005; Hambrick & Oswald, 2005). This ensured attention allocation on both storage and processing components of the task and led us to exclude 18,71 % (N = 64 *participants)*.

In light of the Reviewer's suggestion, which stimulated further interest, we conducted an additional analysis to determine whether the moderation effect observed with the storage component would also appear with the processing component. The analysis was performed on the full sample (n = 342), prior to applying the exclusion criterion. However, the model did not support the presence of an interaction between the symmetry rate and the condition on the quiz. Both results and plot are provided in Table S1 and Figure S3 of the Supplementary Materials.

This is now indicated on page 18 of the manuscript: “The results of a supplementary moderation analysis with the processing component of the WM task are also reported in Table S1 and Figure S3 in the online supplementary material.”

**6. PLOS authors have the option to publish the peer review history of their article (what does this mean?).** If published, this will include your full peer review and any attached files. If you choose “no”, your identity will remain anonymous but your review may still be made public. Do you want your identity to be public for this peer review? For information about this choice, including consent withdrawal, please see our Privacy Policy.

Reviewer #1: Yes: Benito León del Barco

Reviewer #2: No
