# Supplementary material for: Uncovering the relationship between working memory and performance in the Jigsaw classroom
Source: PLoS One. 2025 Mar 18;20(3):e0319495. doi: 10.1371/journal.pone.0319495 (PMC11918379; doi:10.1371/journal.pone.0319495)
Supplement: Attachment — Submitted filename: Response_to_Reviewers_auresp_2.docx [file pone.0319495.s004.docx]

**6. Review Comments to the Author**

Please use the space provided to explain your answers to the questions above. You may also include additional comments for the author, including concerns about dual publication, research ethics, or publication ethics. (Please upload your review as an attachment if it exceeds 20,000 characters)

**Reviewer #2:** Thank you for addressing my concerns. In my opinion this paper is interesting and thought provoking.

We would like to thank you again for taking the time to read our manuscript and the comments we addressed.

**Reviewer #3:** I have read the paper and the comments from a previous round of reviews. I enjoyed reading the paper which I think is well written, and I think the authors have done a good job of addressing the reviewer comments. I only have one comment that I think does need to be addressed before publication.

I think the authors need to exercise more caution in their conclusion that WM mediates the effect of the Jigsaw classroom. I think this for two reasons.

1. I appreciate that the authors have justified their use of the index approach to mediation. But I think there are still reasonable arguments for why you would not look for a mediation effect without a direct effect. I think it is fine to use the index approach, given there clearly are papers to back up this method. But I do think it might necessitate a bit more caution in their interpretation.

2. Much more importantly, the authors run the mediation analysis twice. Once with the original pre-registered measure of WM (pre-experiment) and one using the measure taken during the experiment which was intended to be used for the moderation analysis. I appreciate that the authors have been entirely transparent in this, and have explained and justified why they have done this, and I welcome that. However, their conclusion then states that WM is found to mediate the effect of the Jigsaw classroom. And what they have in fact found is that one analysis does show a mediation effect, and one doesn't. Again, I appreciate they suggest that the second measure of WM is more reflective of the 'real' WMC due to possible anxiety re: the original test and unfamiliarity with the test. Whilst I am sympathetic to a degree with those arguments, the test they used is designed to be administered in one sitting as a valid measure of WM. And so, I think there needs to be more acknowledgement that, overall, their results suggest there *may* be a mediating role for WM, but that it is not conclusive. And I think that needs changing in the abstract and in the Discussion.

We would like to thank you for the review of our manuscript and your comments. We would like to address one particular point in order to ensure the clarity of our manuscript. With regard to your statement 'I think the authors need to exercise more caution in their conclusion that WM mediates the effect of the Jigsaw classroom' and your first remark, it is important to clarify that we did not observe any mediation effect in our study. Although the mediation hypothesis was tested, the model resulted in an absence of indirect and total effect on the student's performance. This is indicated on page 11 in the Results section and in the Discussion section on page 19. However, we did observe that the effects of Jigsaw cooperative learning were moderated by the working memory capacity, leading to better performance among students with lower WMC. This effect was observed on the retest WM score, set as moderator (see pages 17-18 and Figure 1). For this reason, we concluded that our moderation hypothesis was supported by the data. We agree that nevertheless, these findings, as any other work, must call further replication and confirmation. We therefore nuanced our statement and rephrased our claim in the Discussion section. You can see the changes made on pages 20 and 23: ‘Our results suggest that the Jigsaw classroom benefited low WM students, without impacting high WM students’ performance’ and ‘The Jigsaw classroom seems to benefit students with WM limitations, who are often at risk for educational underachievement (e.g., Dunning et al., 2013)’. And on the Abstract on page 3: ‘Multilevel analyses revealed that working memory capacity moderated—but did not mediate—the effect of the Jigsaw classroom. That is, Jigsaw enhanced performance for students with low working memory capacities’.
